# *Chlorella* and *Arthrospira* (Spirulina) as Multi-Pathway Biological Response Modulators: Molecular Mechanisms, Signaling Pathways and Clinical Evidence

**DOI:** 10.3390/molecules31101595

**Published:** 2026-05-10

**Authors:** Wojciech Rzeski, Weronika Rzeska

**Affiliations:** 1 Department of Functional Anatomy and Cytobiology, Institute of Biological Sciences, Maria Curie-Skłodowska University, Akademicka 19, 20-033 Lublin, Poland; 2 Doctoral School of Medical Sciences, Lublin Medical University, 20-093 Lublin, Poland; weronikarzeska@gmail.com

**Keywords:** *Chlorella*, *Arthrospira*, spirulina, phycocyanin, aquatic microalgae, oxidative stress, Nrf2, nuclear factor kappa B (NF-κB), AMPK, nutraceuticals

## Abstract

*Chlorella* and *Arthrospira* (spirulina) are aquatic microalgae of increasing nutraceutical interest due to their dense bioactive composition and multi-target biological activity. This narrative review provides a comparative, mechanistically integrated synthesis of molecular mechanisms and clinical evidence related to their supplementation. Current data indicate that both microalgae converge on three central regulatory axes: activation of Nrf2-dependent antioxidant responses, attenuation of NF-κB-driven inflammatory signaling, and modulation of AMP-activated protein kinase (AMPK)/protein kinase B (AKT)-mediated metabolic pathways. Spirulina demonstrates stronger mechanistic links to intracellular signaling and more consistent clinical evidence for improvements in lipid profile, insulin sensitivity, and systemic inflammation. In contrast, chlorella provides complementary effects, particularly in antioxidant capacity, blood pressure regulation, gut microbiota modulation, and detoxification-related contexts. Their bioactive components—including phycocyanin, carotenoids, polysaccharides, and peptides—act synergistically to influence mitochondrial function, immune homeostasis, and metabolic resilience. While clinical findings are generally consistent, heterogeneity in study design and product standardization limits definitive conclusions. Overall, chlorella and spirulina emerge as complementary multi-pathway biological response modulators with potential applications in preventive and integrative medicine.

## 1. Introduction

Microalgae represent a diverse group of aquatic photosynthetic microorganisms and a rich source of bioactive compounds with significant potential in functional foods, nutraceuticals, and preventive medicine. Among them, species belonging to the genera *Chlorella* and *Arthrospira* (commercially known as spirulina) have attracted increasing scientific and clinical interest due to their dense bioactive composition and broad biological activity. Over the past decade, an expanding body of mechanistic and clinical studies has shifted the perception of these organisms from simple dietary supplements toward complex biological systems capable of modulating redox homeostasis, inflammatory signaling, metabolic regulation, immune responses, and cellular aging processes.

Species such as *Chlorella vulgaris* and *Chlorella pyrenoidosa* are unicellular freshwater and aquatic green microalgae characterized by high chlorophyll content, carotenoids, bioactive peptides, polysaccharides, vitamins, and the so-called *Chlorella* Growth Factor (CGF), a water-soluble extract rich in nucleotides and small peptides. In parallel, the cyanobacteria *Arthrospira platensis* and *Arthrospira maxima* (commonly referred to as spirulina) are distinguished by their high protein content and unique pigments such as phycocyanin, along with sulfated polysaccharides, essential fatty acids (including γ-linolenic acid), and diverse phenolic compounds [1,2,3].

Both *Chlorella* and *Arthrospira* contain structurally and functionally diverse classes of bioactive molecules that act on multiple molecular targets. Rather than exerting a single dominant pharmacological effect, these microalgae influence interconnected signaling networks involved in redox balance, inflammation, and metabolic regulation. This multimodal activity aligns with current systems-biology approaches to chronic disease prevention, where the modulation of network dynamics is considered more effective than targeting isolated molecules [2,4,5,6].

Throughout this review, the term “spirulina” refers to commercial preparations derived primarily from *Arthrospira platensis* or *A. maxima*, consistent with established nutraceutical and clinical literature, while “*Arthrospira*” denotes the organism at the species level.

Emerging evidence suggests that supplementation with *Chlorella* and spirulina may be associated with improvements in metabolic parameters such as insulin sensitivity, lipid profiles, and hepatic steatosis markers while simultaneously reducing systemic inflammatory burden and oxidative stress. In parallel, emerging data indicate potential roles in immunomodulation, gut microbiota regulation, endothelial protection, and even chemoprevention. Of particular interest is the hypothesis that these organisms may contribute to “metabolic resilience” and healthy aging by attenuating chronic low-grade inflammation (inflammaging), supporting mitochondrial integrity, and enhancing endogenous antioxidant defenses [5,7,8,9,10].

Clinical meta-analyses report significant improvements in cardiometabolic markers with spirulina supplementation: total cholesterol reductions of approximately 0.4–1.0 mmol/L, low-density lipoprotein cholesterol (LDL-C) reductions of 0.2–0.8 mmol/L, triglyceride reductions of 0.3–0.6 mmol/L, and decreases in C-reactive protein (CRP) of 0.5–1.5 mg/L, particularly in populations with pre-existing cardiometabolic risk factors [4,7,8,9].

Despite the rapidly expanding literature, the field remains fragmented. Many studies focus on isolated outcomes without integrating molecular mechanisms with clinical endpoints. Additionally, variability in cultivation conditions, extraction methods, strain specificity, and product standardization complicates comparison across studies. There is therefore a need for a comprehensive and mechanistically integrated synthesis of current knowledge that bridges biochemical composition, cellular signaling pathways, preclinical models, and human clinical trials [5,11,12,13].

The aim of the present review is to provide a comparative and mechanistically integrated overview of the biological activities of *Chlorella* and *Arthrospira* (spirulina), with particular emphasis on antioxidant, anti-inflammatory, metabolic, immunomodulatory, and potential anti-aging effects. We also discuss clinical evidence, safety considerations, and translational perspectives, highlighting current knowledge gaps and future research directions necessary for the rational development of microalgae-based interventions in preventive and integrative medicine.

Importantly, this review goes beyond descriptive synthesis and proposes an integrative conceptual framework in which chlorella and spirulina act as multi-axis regulators of the redox–inflammation–metabolism network, rather than isolated nutraceutical agents.

## 2. Chemical Composition and Bioactive Constituents

The biological activity of microalgae such as *Chlorella* and *Arthrospira* (spirulina) is largely determined by their complex biochemical composition. These organisms contain diverse classes of bioactive molecules, including proteins, pigments, polysaccharides, lipids, vitamins, minerals, and small regulatory compounds. Rather than acting through a single dominant constituent, their physiological effects appear to emerge from the combined activity of multiple compounds that influence interconnected cellular pathways. Understanding the chemical composition of these microalgae therefore provides an essential foundation for interpreting their antioxidant, anti-inflammatory, metabolic, and immunomodulatory properties, as discussed in the following sections [1,2,3].

### 2.1. Major Bioactive Constituents

Both *Chlorella* and *Arthrospira* are aquatic photosynthetic microorganisms cultivated in freshwater and alkaline lake environments. Commercial “spirulina” corresponds mainly to *Arthrospira platensis*/*A. maxima* (cyanobacteria, native to tropical alkaline lakes), while chlorella typically refers to *Chlorella vulgaris*/*C. pyrenoidosa* (unicellular freshwater green microalgae). Their biological activity depends strongly on the product form: whole dried biomass (tablet/powder), disrupted-cell products, hot-water extracts, enzymatic hydrolysates, or purified fractions (e.g., phycocyanin). This variability likely contributes to the heterogeneity observed across clinical trials and meta-analyses [1,2,11,14].

#### 2.1.1. Proteins and Bioactive Peptides

Both organisms are protein-dense, but spirulina typically has a higher protein fraction and a rich spectrum of peptides released during digestion or enzymatic hydrolysis. These peptides are implicated in antioxidant effects (including reactive oxygen species (ROS) scavenging and support of endogenous antioxidant enzymes), anti-inflammatory signaling modulation via NF-κB-related pathways, metabolic effects related to insulin signaling and substrate utilization, and potential ACE-inhibitory and endothelial-supporting actions reported in the broader algae peptide literature [1,2,3,7,15].

A key spirulina-derived protein complex is C-phycocyanin (and derived peptides), which is repeatedly linked with redox and inflammatory pathway modulation (Nrf2/heme oxygenase-1 (HO-1); NF-κB), providing a mechanistic bridge between preclinical and translational observations [2,16,17].

#### 2.1.2. Pigments (Chlorophylls, Carotenoids, Phycobiliproteins)

*Chlorella* derives much of its antioxidant activity from chlorophylls and carotenoids (including lutein and β-carotene), which contribute to free radical neutralization and membrane protection [1,18].

In spirulina, phycobiliproteins, particularly phycocyanin, are the dominant pigment-derived bioactives, with documented antioxidant and anti-inflammatory activity and emerging evidence for metabolic signaling effects including Nrf2 activation and the attenuation of oxidative stress in preclinical models [2,7,19].

#### 2.1.3. Polysaccharides and Cell-Wall Related Bioactivity

Polysaccharides (including sulfated polysaccharides more typical for cyanobacteria-derived fractions) are often discussed as mediators of immunomodulation (innate immune tuning, cytokine response shaping), gut barrier and microbiota interactions (prebiotic-like effects and barrier support hypotheses), and anti-inflammatory effects (including in gut-associated models) [1,2,20].

In spirulina, polysaccharide-rich fractions are commonly connected to immune activity; in chlorella, cell-wall composition and degree of cell disruption (mechanical/enzymatic) can materially affect bioaccessibility and downstream biological readouts [1,21,22].

#### 2.1.4. Lipid Fraction and Fatty Acids

While total lipid content is not the main “headline” feature versus proteins/pigments, spirulina is notable for γ-linolenic acid (GLA) and a lipid profile that may contribute to anti-inflammatory balance and metabolic endpoints (especially when supplementation is combined with lifestyle interventions in clinical contexts). Clinical meta-analyses in overweight/obesity settings often discuss lipids and inflammation jointly as outcomes rather than isolating single molecules [2,4,7].

#### 2.1.5. Nucleotides, Small Molecules, and *Chlorella* Growth Factor (CGF)

A distinctive concept in the chlorella literature is *Chlorella* Growth Factor (CGF), a water-soluble extract fraction described as nucleotide- and peptide-rich, and frequently summarized as containing small peptides, amino acids, nucleotides, glycoproteins, vitamins, and minerals. Importantly, recent technical reviews emphasize that the CGF composition is not standardized and depends on species/strain, culture conditions, and extraction process, which is crucial for translational reproducibility [1,11].

### 2.2. Quality, Standardization, and Contamination

Because both spirulina and chlorella can bioaccumulate heavy metals and may be susceptible to cyanotoxin contamination (e.g., microcystins via co-occurring cyanobacteria), a modern review should integrate quality-control issues into the interpretation of efficacy and safety outcomes. This is now treated as a mainstream consideration in the literature, and not a marginal “limitations” note [11,12]. The main compositional differences between chlorella and spirulina are summarized in Table 1.

In clinical trials, spirulina and chlorella have generally demonstrated a favorable safety profile at commonly used doses (1–10 g/day), with adverse effects typically limited to mild gastrointestinal symptoms. Potential drug interactions (e.g., with anticoagulants due to vitamin K content) and contraindications in phenylketonuria (phenylalanine content) should be noted [11,12].

## 3. Antioxidant and Redox-Modulating Activity

Oxidative stress is a central pathophysiological mechanism underlying metabolic disorders, cardiovascular disease, neurodegeneration, and aging-related functional decline. Reactive oxygen species (ROS) are not merely harmful by-products of metabolism but also key signaling mediators; thus, effective intervention strategies should aim at redox modulation rather than indiscriminate radical scavenging. Accumulating evidence suggests that *Chlorella* and *Arthrospira* exert multi-layered antioxidant effects involving direct radical neutralization, metal chelation, enhancement of endogenous antioxidant defenses, and mitochondrial protection [4,5,23].

### 3.1. Activation of the Nrf2/Keap1 Pathway

One of the most consistently reported mechanisms involves activation of the Nrf2 pathway. Nrf2 regulates the transcription of genes encoding antioxidant and cytoprotective enzymes, including heme oxygenase-1 (HO-1), superoxide dismutase (SOD), catalase, and glutathione peroxidase (GPx) [17,24,25].

At the monomeric level, specific compounds have been studied for their individual pharmacological effects. C-phycocyanin, the major pigment-protein of *Arthrospira*, acts as a potent Nrf2 activator and NF-κB inhibitor through its chromophore phycocyanobilin, a redox-active molecule that modulates cellular thiol status [3,18]. Specific polysaccharide fractions (e.g., CPP-3a from *Chlorella pyrenoidosa*) activate macrophages via TLR4 engagement, while sulfated polysaccharides from *Arthrospira* exhibit anti-inflammatory activities in experimental models [2,20].

Phycocyanin and phycocyanin-derived peptides from *Arthrospira platensis* have been shown in preclinical models to promote Nrf2 nuclear translocation and upregulate HO-1 expression, leading to reduced lipid peroxidation and improved mitochondrial integrity. Similarly, carotenoids and chlorophyll derivatives from *Chlorella vulgaris* have demonstrated the capacity to enhance endogenous antioxidant enzyme activity and attenuate oxidative tissue damage in various in vivo models [17,24,26,27,28].

This mechanism suggests that microalgae supplementation may support adaptive antioxidant responses, aligning with the concept of hormetic redox regulation rather than simple antioxidant supplementation [4,5,29].

### 3.2. Inhibition of NF-κB and Redox–Inflammation Crosstalk

Redox homeostasis and inflammatory signaling are tightly interconnected. Excess ROS can activate nuclear factor kappa B (NF-κB), which in turn amplifies inflammatory cytokine production (e.g., interleukin-6 (IL-6), tumor necrosis factor alpha (TNF-α)), further increasing oxidative stress [17,30,31].

Both chlorella and spirulina preparations have been reported to attenuate NF-κB activation in experimental models. Phycocyanin appears particularly active in modulating this redox–inflammation axis, reducing oxidative stress markers while simultaneously lowering pro-inflammatory mediators. This dual modulation is especially relevant in conditions characterized by chronic low-grade inflammation (inflammaging, metabolic syndrome, non-alcoholic fatty liver disease (NAFLD)) [15,26,30,32].

Clinical studies frequently report improvements in oxidative stress markers such as malondialdehyde (MDA) and improvements in total antioxidant capacity (TAC), often accompanied by decreased CRP or pro-inflammatory cytokines, although the magnitude of effects varies across populations and study designs. While causality at the molecular level in humans remains to be fully clarified, the alignment of mechanistic and clinical observations strengthens the mechanistic rationale for these effects [18,19,33,34,35].

### 3.3. Mitochondrial Protection and Bioenergetics

Mitochondria are both major sources and primary targets of ROS. Age-related mitochondrial dysfunction contributes to metabolic inflexibility, insulin resistance, endothelial dysfunction, and neurodegenerative processes [4,5,36].

Experimental evidence suggests that bioactive compounds from spirulina and chlorella may reduce mitochondrial ROS generation, preserve mitochondrial membrane potential, enhance ATP production efficiency, and support mitophagy and mitochondrial quality control, as suggested in emerging models.

By improving mitochondrial redox balance, microalgae-derived compounds may indirectly enhance metabolic resilience and cellular longevity pathways. These observations are particularly relevant in the context of aging and metabolic disease, where mitochondrial dysfunction is a core feature [17,27,37,38,39,40].

### 3.4. Clinical Evidence of Redox Modulation

Randomized controlled trials investigating spirulina supplementation in populations with metabolic syndrome, obesity, or type 2 diabetes frequently report decreased lipid peroxidation markers (e.g., MDA), increased SOD and GPx activity, and improved total antioxidant status. *Chlorella* supplementation trials similarly indicate improvements in oxidative stress biomarkers, and in some cohorts, blood pressure and endothelial function, suggesting that redox modulation may contribute to vascular benefits [8,9,41,42]. These results must be interpreted with caution. Dosages ranged from 1 to 8 g/day across trials, durations varied from 4 to 24 weeks, and no standardized extract definition currently exists—three sources of heterogeneity that make cross-study comparison inherently difficult. Additionally, most trials lack mechanistic biomarkers capable of confirming the proposed signaling pathways in humans. Thus, while clinical data are encouraging, the magnitude of observed effects is generally moderate, and methodological limitations including heterogeneity of interventions and insufficient mechanistic biomarkers in humans reduce the certainty of causal interpretation [7,9,15,43]. It should be emphasized that the magnitude of these effects is generally moderate, and methodological limitations including heterogeneity of interventions and insufficient mechanistic biomarkers in human studies limit the strength of causal inference.

### 3.5. Redox Modulation in the Context of Healthy Aging

Chronic oxidative stress is a hallmark of aging and contributes to genomic instability, telomere attrition, mitochondrial dysfunction, and cellular senescence. By enhancing endogenous antioxidant defenses and modulating inflammatory signaling, chlorella and spirulina may attenuate aspects of “inflammaging” and oxidative damage accumulation [4,5,44].

Although long-term aging trials in humans are lacking, the combined antioxidant, anti-inflammatory, and metabolic effects suggest a plausible role for microalgae supplementation as part of a broader strategy aimed at promoting metabolic resilience and healthy lifespan [5,45,46,47,48,49]. Representative clinical trials investigating the cardiometabolic and antioxidant effects of chlorella and spirulina supplementation are summarized in Table 2.

Overall, clinical evidence indicates that spirulina supplementation improves lipid profiles and reduces oxidative stress markers across diverse populations, including individuals with dyslipidemia, metabolic syndrome, and type 2 diabetes. *Chlorella* supplementation appears particularly promising in blood pressure modulation and improvement of antioxidant capacity. However, substantial heterogeneity in dosage, duration, and preparation type remains a key limitation affecting cross-study comparability.

## 4. Anti-Inflammatory and Immunomodulatory Effects

Chronic low-grade inflammation is a key driver of cardiometabolic disease, insulin resistance, endothelial dysfunction, and age-related decline. Unlike acute inflammatory responses, persistent activation of innate immune pathways, particularly those involving NF-κB, mitogen-activated protein kinase (MAPK) signaling, and inflammasome activation, contributes to systemic metabolic dysregulation. Increasing mechanistic and clinical evidence indicates that both *Chlorella* and *Arthrospira* (spirulina) modulate inflammatory signaling at multiple regulatory levels [4,5,33,34,50].

### 4.1. Modulation of NF-κB and Pro-Inflammatory Mediators

The transcription factor NF-κB represents a central node linking oxidative stress to inflammatory gene expression. In macrophage models stimulated with lipopolysaccharide (LPS), C-phycocyanin derived from *Arthrospira platensis* reduced inducible nitric oxide synthase (initric oxide (NO)S) expression, nitric oxide production, and pro-inflammatory mediators. More recent work further indicates that phycocyanin modulates upstream signaling cascades including MAPK (ERK, JNK, p38) and IκB phosphorylation, thereby limiting NF-κB nuclear translocation [27,30,38,51].

Similarly, fractions derived from *Chlorella vulgaris* have been shown to suppress inflammatory mediators in cellular models by reducing NO production and downregulating cyclooxygenase-2 (COX-2) and iNOS expression. In toxicant-induced animal models, chlorella supplementation attenuates NF-κB activation while simultaneously enhancing Nrf2-mediated antioxidant defense, highlighting the integrated redox–inflammation axis [15,26].

Overall, the available evidence supports a model of coordinated signaling modulation: microalgae-derived compounds do not simply neutralize ROS but reset inflammatory gene expression at its transcriptional source—a distinction with important implications for chronic disease contexts where redox and immune dysregulation are causally intertwined.

### 4.2. Macrophage Polarization and Innate Immunity

Macrophage plasticity plays a crucial role in chronic inflammatory disorders. Recent mechanistic studies on *Chlorella pyrenoidosa* polysaccharides (e.g., CPP-3a) demonstrate activation of the TLR4/2–MyD88–NF-κB and p38 MAPK pathways, leading to regulated macrophage activation and cytokine modulation. Notably, polysaccharide structure appears to determine immunomodulatory potency, supporting a structure–function relationship [52].

In vivo models further indicate that spirulina and chlorella supplementation influence immune cell balance, including the modulation of T-cell subsets and anti-inflammatory cytokine expression. In murine colitis models, chlorella biomass supplementation altered gut microbiota composition, increased short-chain fatty acid production, and promoted regulatory T-cell (Treg) responses, resulting in the attenuation of inflammatory pathology. These findings position chlorella not only as a systemic anti-inflammatory agent but also as a gut–immune axis modulator [21,53]. Importantly, clinical evidence in healthy volunteers demonstrates that oral administration of a hot water extract of *Spirulina platensis* augmented natural killer (NK) cell cytotoxicity and IFN-γ production in an IL-12/IL-18-dependent manner, with effects on both NK cells and monocyte/macrophage lineages, suggesting that spirulina acts preferentially on the innate immune arm rather than adaptive immunity [54]. Parallel clinical evidence from a randomized, double-blind, placebo-controlled trial showed that 8 weeks of *Chlorella* supplementation (5 g/day) in healthy individuals significantly increased NK cell cytotoxic activity and elevated serum IFN-γ and IL-1β concentrations, with the magnitude of NK cell changes positively correlating with Th-1 cytokine levels [55]. This NK-potentiating effect represents one of the few immunomodulatory outcomes directly confirmed in human subjects for both organisms, and underscores a clinically relevant dimension of their biological response modifier properties. In an in vitro study directly relevant to cancer immunology, chlorella extract did not compromise the viability of NK-92 cells while exerting cytotoxic effects on human colon cancer cells, supporting a selective anti-tumor immune context for chlorella-associated immunomodulation [56].

### 4.3. Systemic Inflammation and Clinical Biomarkers

Clinical trials investigating spirulina supplementation in individuals with metabolic syndrome, obesity, or type 2 diabetes frequently report reductions in circulating C-reactive protein (CRP), with variable effects on IL-6 and TNF-α. Meta-analyses of randomized controlled trial (RCT)s indicate a statistically significant reduction in CRP following spirulina supplementation, while broader evidence syntheses published in 2025 also support favorable effects on inflammatory and oxidative-stress biomarkers [33,34,57,58]. Nevertheless, heterogeneity across studies, differences in baseline inflammatory status, and the possibility of publication bias should be considered when interpreting these findings. *Chlorella* supplementation has similarly been associated with reductions in inflammatory and oxidative stress biomarkers in populations with metabolic dysfunction and prehypertension, often accompanied by improvements in antioxidant enzyme activity. While heterogeneity remains considerable across studies, convergence between mechanistic and clinical evidence strengthens the mechanistic basis for anti-inflammatory action. Despite these findings, variability in baseline inflammatory status, study design, and intervention standardization introduces substantial heterogeneity, and therefore the clinical relevance of observed reductions in inflammatory markers should be interpreted cautiously.

### 4.4. Interaction Between Redox and Immune Signaling

An important conceptual aspect is the bidirectional interaction between oxidative stress and immune activation. Activation of Nrf2 signaling can indirectly suppress inflammatory transcriptional activity, while the inhibition of NF-κB reduces ROS amplification loops. Several studies in both spirulina and chlorella models demonstrate concurrent activation of Nrf2/HO-1 and suppression of NF-κB pathways, suggesting coordinated redox–immune reprogramming rather than isolated pathway inhibition [17,39,59].

This bidirectional modulation is particularly relevant in conditions characterized by “inflammaging”, where persistent immune activation and oxidative stress coexist. By targeting upstream signaling convergence points, microalgae-derived bioactives may contribute to the restoration of immune homeostasis.

### 4.5. Translational Implications

*Chlorella* and spirulina are not simply anti-inflammatory agents. Their immune effects shift with biological context: suppressing excessive inflammatory activation while preserving—or in some experimental settings enhancing—baseline immune vigilance. This adaptability is consistent with the concept of biological response modulation and distinguishes these organisms from conventional immunosuppressive nutraceuticals. In vitro data indicate the capacity to attenuate excessive inflammatory responses, while certain polysaccharide fractions may enhance innate immune vigilance under non-pathological conditions. This bidirectional adaptability is consistent with the concept of biological response modulation rather than simple anti-inflammatory suppression [17,21,39,52,60].

However, several limitations should be acknowledged: variability in strain, cultivation, and extraction methods, limited integration of immunophenotyping in clinical trials, and insufficient long-term human data evaluating immune aging outcomes.

Future studies combining randomized supplementation trials with transcriptomic, proteomic, and immunophenotyping approaches will be essential to define precise mechanisms in humans.

Key mechanistic studies supporting these pathways are presented in Table 3.

## 5. Metabolic Effects: Insulin Sensitivity, Lipid Homeostasis, NAFLD, and Metabolic Resilience

Metabolic regulation represents one of the key physiological domains influenced by microalgae-derived bioactive compounds, linking redox balance, inflammatory signaling, and energy metabolism [4,5,7]. Metabolic dysfunction, including insulin resistance, dyslipidemia, visceral adiposity, and non-alcoholic fatty liver disease (NAFLD), is closely intertwined with chronic inflammation and oxidative stress. Given the multi-target biological activity of *Chlorella* and *Arthrospira* (spirulina), increasing attention has been directed toward their potential role in modulating core metabolic pathways at both molecular and clinical levels. Rather than acting as isolated hypoglycemic or hypolipidemic agents, these microalgae appear to influence interconnected signaling networks involved in energy sensing, mitochondrial function, inflammation, and lipid metabolism [4,5,7,8,9,64].

Preclinical studies suggest that spirulina-derived phycocyanin can modulate insulin signaling pathways, including AKT phosphorylation and AMPK activation, thereby improving glucose uptake and reducing insulin resistance in diet-induced metabolic models. In murine models of high-fat/high-glucose feeding, phycocyanin supplementation has been associated with improved insulin sensitivity indices, reduced hepatic lipid accumulation, and attenuation of oxidative stress markers. These effects are often accompanied by the activation of AMPK, a master regulator of cellular energy homeostasis that enhances fatty acid oxidation, suppresses lipogenesis, and improves mitochondrial efficiency. Importantly, AMPK activation also exerts anti-inflammatory effects via the indirect inhibition of NF-κB signaling, further linking metabolic and immune regulation [37,65]. Of particular mechanistic relevance, AMPK activation simultaneously suppresses mammalian target of rapamycin (mTOR) signaling, thereby promoting autophagy and mitophagy—processes central to cellular quality control, mitochondrial turnover, and healthy aging. This AMPK–mTOR axis therefore links the metabolic effects of spirulina supplementation to broader cellular homeostasis pathways.

*Chlorella* supplementation in experimental models has similarly demonstrated protective effects against diet-induced metabolic derangements. In rodent models of hepatic steatosis, chlorella biomass or extracts reduced triglyceride accumulation in the liver, improved antioxidant enzyme activity, and attenuated inflammatory signaling. Mechanistically, these effects have been associated with the modulation of lipid metabolism-related gene expression, suppression of oxidative stress-driven inflammatory cascades, and improvement in mitochondrial integrity. The capacity of chlorella to influence both redox balance and lipid handling supports a model in which metabolic benefits arise from the coordinated regulation of oxidative and inflammatory stressors rather than from direct lipid-lowering action alone [26,41,66].

Beyond intracellular signaling, the gut–liver axis appears to represent an additional mechanism underlying metabolic effects. Animal studies have demonstrated that chlorella supplementation can reshape gut microbiota composition, increase short-chain fatty acid (SCFA) production, and promote regulatory immune responses, resulting in attenuation of colitis and systemic inflammation. Given the established role of gut dysbiosis in metabolic syndrome and NAFLD, microbiota-mediated modulation may represent a critical component of microalgae-induced metabolic resilience. Spirulina-derived compounds have also been shown to influence gut microbial composition in experimental models, although human data remain limited and heterogeneous [21,63,67].

Clinical evidence further supports metabolic benefits, particularly for spirulina. Multiple randomized controlled trials and meta-analyses indicate consistent reductions in total cholesterol (approximately 10–20 mg/dL), LDL-C (approximately 8–15 mg/dL), and triglycerides (approximately 15–30 mg/dL), with modest increases in HDL-C across diverse populations. Improvements in fasting blood glucose and indices of insulin resistance (e.g., homeostatic model assessment of insulin resistance, HOMA-IR) have been observed in individuals with type 2 diabetes and metabolic syndrome, especially with doses ≥2 g/day administered for at least 8–12 weeks. Reductions in systemic inflammation markers such as CRP often accompany these metabolic improvements, reinforcing the concept of integrated metabolic–inflammatory modulation [7,18,19,33,34,48,57,68].

*Chlorella* supplementation has shown promising effects on blood pressure, glycemic control, and lipid parameters in individuals with prehypertension and metabolic dysfunction. Some studies report improvements in liver enzyme profiles in NAFLD patients, suggesting potential hepatoprotective action. However, the number of high-quality RCTs remains smaller compared to spirulina, and greater standardization of preparations is needed to draw definitive conclusions [8,9,15,41,69].

Metabolic benefits are unlikely to be solely attributable to a single compound class. Phycocyanin, polysaccharides, carotenoids, bioactive peptides, and micronutrients likely act synergistically. For example, antioxidant effects may reduce oxidative impairment of insulin receptor signaling; anti-inflammatory activity may attenuate adipose tissue macrophage activation; and AMPK activation may promote mitochondrial biogenesis and lipid oxidation. Such coordinated network modulation aligns with systems-biology perspectives on metabolic disease, where the restoration of homeostatic balance requires multi-target intervention [1,2,4,5,37,70].

In the context of NAFLD, where oxidative stress, lipotoxicity, mitochondrial dysfunction, and inflammation converge, the wide-ranging properties of spirulina and chlorella are particularly relevant. Preclinical evidence supports reductions in hepatic lipid accumulation and improvements in oxidative injury markers, while limited human data suggest possible improvement in liver enzymes and cardiometabolic risk factors. Nevertheless, long-term trials with imaging-based endpoints and histological confirmation are still lacking [9,15,41,71].

Taken together, these findings support a model in which microalgae influence metabolic regulation through the coordinated modulation of energy sensing, redox balance, and inflammatory signaling. *Chlorella* and *Arthrospira* may therefore enhance metabolic resilience through the coordinated modulation of insulin signaling, redox homeostasis, inflammatory tone, and gut microbiota composition. While the magnitude of clinical effects is generally moderate, the consistency across lipid and inflammatory endpoints suggests biological relevance. Future studies incorporating metabolomics, transcriptomics, and standardized extract characterization will be essential to clarify dose–response relationships and identify responder phenotypes [5,37,48,57,72]. These metabolic mechanisms interact with broader systemic pathways, influencing cardiovascular and mitochondrial function, as discussed in the following section.

## 6. Systems-Level Integration: Mitochondrial Function, Cardiovascular Protection and Neurovascular Signaling

The biological activity of *Chlorella* and *Arthrospira* extends beyond isolated antioxidant or metabolic effects and converges at the level of mitochondrial regulation, vascular homeostasis, immune signaling, and cellular stress adaptation. Chronic diseases such as cardiovascular disease, metabolic syndrome, non-alcoholic fatty liver disease (NAFLD), and neurodegenerative disorders share common upstream drivers, including oxidative stress, low-grade inflammation, mitochondrial dysfunction, and impaired nutrient sensing. Within this context, microalgae-derived bioactive compounds appear to act as multi-target modulators influencing interconnected regulatory networks.

**Figure 1:**
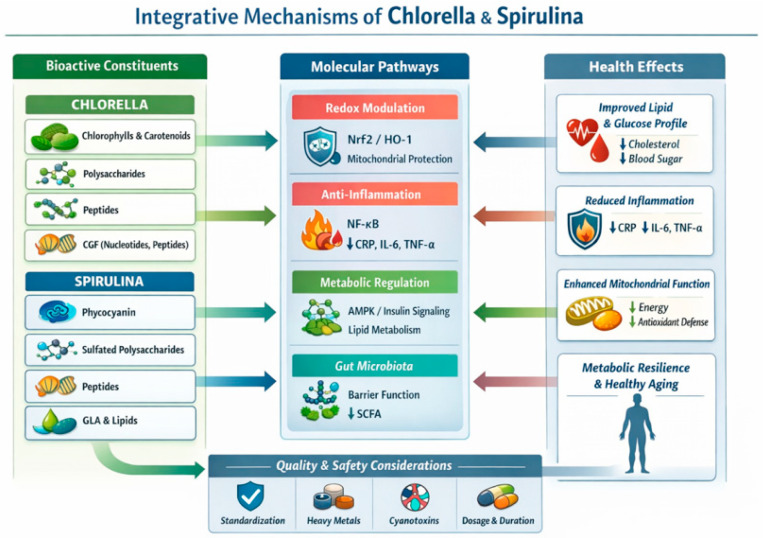
Integrative mechanisms of biological activity of *Chlorella* and *Arthrospira* (spirulina). Bioactive compounds derived from chlorella and spirulina modulate interconnected regulatory pathways involved in oxidative stress control, inflammatory signaling, metabolic regulation, and mitochondrial function. Spirulina-derived components, particularly phycocyanin and related pigments, are associated with the activation of Nrf2-dependent antioxidant responses, inhibition of NF-κB-mediated inflammatory signaling, and modulation of energy-sensing pathways such as AMPK and AKT. These mechanisms contribute to improved lipid metabolism, reduced systemic inflammation, and enhanced metabolic resilience. *Chlorella*-derived bioactives, including chlorophylls, carotenoids, and structural polysaccharides, support antioxidant defense systems, modulate immune signaling, and influence gut microbiota composition, potentially increasing short-chain fatty acid production and improving gut–immune interactions. Figure created by the authors.

The interactions between redox regulation (Nrf2), inflammatory signaling (NF-κB), and metabolic control (AMPK/AKT) form an integrated network, as illustrated in Figure 1. These pathways do not operate independently but constitute a tightly interconnected system in which modulation of one axis influences the others. From a systems-level perspective, these findings support the concept that the observed biological activity is not a simple sum of independent effects, but rather reflects coordinated pathway crosstalk.

### 6.1. Mitochondrial Regulation and Cellular Energy Sensing

Mitochondria represent a central hub in metabolic and aging-related pathophysiology. Excessive reactive oxygen species production, impaired oxidative phosphorylation, and defective mitochondrial quality control contribute to metabolic inflexibility and tissue dysfunction [4,5,36]. Experimental evidence suggests that bioactive compounds derived from spirulina, particularly phycocyanin, may reduce mitochondrial ROS generation, preserve mitochondrial membrane potential, and enhance endogenous antioxidant defenses through the activation of Nrf2-dependent pathways [17,27,37,38,39,40].

In addition, spirulina supplementation in metabolic disease models has been associated with the activation of AMPK, a key regulator of cellular energy homeostasis. This activation promotes a shift toward increased substrate oxidation, reduced lipid synthesis, and improved mitochondrial efficiency. The net effect is enhanced metabolic flexibility under conditions of nutrient excess or oxidative stress, which in preclinical models is associated with reduced ectopic lipid accumulation and improved insulin sensitivity indices [37,65].

Although direct evidence for chlorella-induced mitochondrial biogenesis remains limited, chlorella biomass and extracts consistently demonstrate redox-stabilizing properties. By reducing lipid peroxidation and enhancing antioxidant enzyme activity, chlorella may indirectly support mitochondrial integrity and respiratory function, particularly under inflammatory or toxic stress conditions [26,41,66].

### 6.2. Cardiovascular Protection and Endothelial Function

Cardiovascular protection represents one of the most clinically relevant domains of microalgae supplementation. Oxidative stress and chronic inflammation contribute to endothelial dysfunction, reduced nitric oxide bioavailability, and atherogenic lipid modification [4,5,36]. Spirulina supplementation has been associated with improvements in lipid profiles, including reductions in total cholesterol, LDL cholesterol, and triglycerides, often accompanied by decreases in oxidative stress biomarkers and inflammatory markers such as C-reactive protein [7,18,19,33,34,48,57,68].

These vascular effects likely reflect the integrated modulation of lipid metabolism, redox balance, and inflammatory signaling. Phycocyanin and related compounds may contribute to endothelial protection by limiting oxidative damage and attenuating NF-κB-mediated inflammatory activation, thereby supporting nitric oxide signaling pathways [17,30,38].

*Chlorella* appears to exhibit a somewhat different cardiovascular profile. Clinical studies in prehypertensive populations report reductions in systolic blood pressure (SBP) and improvements in antioxidant capacity following chlorella supplementation. These effects are likely mediated by improved endothelial redox balance and reduced inflammatory burden rather than direct vasodilatory mechanisms [8,9,41,57].

### 6.3. Neurovascular and Neuroimmune Signaling

Emerging evidence suggests that both spirulina and chlorella may influence neurovascular and neuroimmune pathways. Chronic systemic inflammation and metabolic dysfunction are increasingly recognized as important contributors to cognitive decline and neurodegenerative disease [4,5,36]. Experimental studies indicate that phycocyanin can attenuate microglial activation, reduce oxidative neuronal injury, and modulate inflammatory cytokine production within neural tissues [27,38]. In addition, in vivo models provide further support for neuroprotective effects, including the preservation of dopaminergic neurons and reduced neuroinflammation in Parkinson’s disease models, as well as the attenuation of oxidative stress and cognitive decline in experimental models of neurodegeneration [61,73,74]. It should be noted, however, that current evidence in this domain is largely limited to preclinical models, and human clinical data remain insufficient to draw definitive conclusions. Similarly, chlorella-derived polysaccharides have demonstrated neuroprotective properties in experimental models, potentially through the modulation of inflammatory signaling and gut–brain axis interactions [21,63,67].

Changes in gut microbiota composition and short-chain fatty acid production may represent an additional mechanism linking microalgae supplementation with neuroimmune regulation. However, the relevance of these findings in human populations has yet to be established.

### 6.4. Comparative Systems Perspective

From a systems-biology perspective, spirulina and chlorella appear to exert partially overlapping but distinct biological effects. Spirulina shows stronger evidence for the direct modulation of intracellular signaling pathways involved in energy sensing and inflammation, including AMPK, AKT, and NF-κB. These mechanisms are consistent with its more robust clinical evidence in dyslipidemia and metabolic syndrome [7,8,9,33,34,48,57].

In contrast, chlorella appears to exert broader effects at the interface of oxidative stress regulation, gut microbiota modulation, immune signaling, and detoxification-related processes. These characteristics may contribute to its observed effects on blood pressure regulation, antioxidant capacity, and environmental stress responses [8,9,41,57].

Taken together, these observations support a model of pathway complementarity rather than redundancy. Nrf2 activation attenuates NF-κB-driven inflammation, AMPK signaling influences both metabolic and inflammatory pathways, and gut microbiota modulation shapes systemic immune tone [17,39,59]. These interactions are interdependent rather than additive: redox balance influences immune activation, which in turn affects metabolic regulation and mitochondrial function.

*Chlorella* and spirulina therefore appear to engage multiple entry points within this regulatory network simultaneously, which may help explain the breadth and relative consistency of their reported biological effects. This systems-level perspective provides a conceptual framework for understanding their role as multi-pathway biological response modulators rather than single-target nutraceuticals. The integrative mechanisms linking redox regulation, inflammation, metabolic signaling, and systemic health outcomes are illustrated in Figure 1.

A comparative overview of the biological activities and translational strength of chlorella and spirulina is summarized in Table 4.

This systems-level perspective highlights that the biological effects of microalgae supplementation cannot be attributed to single compounds but rather emerge from the coordinated modulation of interconnected metabolic, inflammatory, and redox networks.

## 7. Combined Supplementation of *Chlorella* and *Arthrospira* (Spirulina): Rationale, Evidence, and Practical Considerations

Nutraceutical research increasingly emphasizes multi-component interventions targeting interconnected biological pathways involved in metabolic and inflammatory disorders [1,2,5]. Given partially overlapping yet distinct biochemical profiles, combined supplementation with *Chlorella* and *Arthrospira* (spirulina) is frequently promoted as a strategy to broaden health benefits. From a mechanistic standpoint, such an approach is biologically plausible because the two organisms provide complementary classes of bioactives that may act on different “layers” of physiological regulation.

Spirulina is typically richer in phycobiliproteins, particularly C-phycocyanin and high-quality protein/peptides, and has comparatively stronger mechanistic links to redox–inflammation hubs such as Nrf2/HO-1 and NF-κB, as well as to energy-sensing pathways including AMPK and AKT in preclinical metabolic models. Clinically, spirulina has more consistent evidence for improving lipid profiles and reducing systemic inflammatory burden (often reflected by CRP reductions) in populations with dyslipidemia or metabolic syndrome. These effects can be interpreted as acting primarily through intracellular signaling modulation, improved redox buffering, and downstream metabolic consequences.

*Chlorella*, while also exhibiting antioxidant and anti-inflammatory activity, appears to have distinctive strengths related to chlorophyll/carotenoid content, structural polysaccharides, and gut–immune interactions. Preclinical evidence supports microbiota reshaping, increased short-chain fatty acid (SCFA) production, and regulatory immune responses in inflammatory models. Additionally, chlorella has been repeatedly discussed in the context of detoxification-related effects and pollutant/heavy-metal binding, which may indirectly reduce oxidative and inflammatory stress load. Clinically, chlorella shows a relatively consistent signal for blood pressure reduction and improvements in antioxidant capacity in some cohorts.

On this basis, co-supplementation could theoretically provide broader “systems coverage”: spirulina contributes more strongly to intracellular redox and metabolic signaling (e.g., AMPK/NF-κB modulation), while chlorella supports gut-mediated immune homeostasis and reduces external stressor burden (detox-related contexts), potentially enhancing net metabolic resilience. Such complementarity may be particularly relevant for integrated cardiometabolic risk reduction, where lipid metabolism, inflammation, oxidative stress, gut dysbiosis, and endothelial dysfunction interact.

However, despite strong mechanistic plausibility, the clinical evidence base for combined supplementation remains limited. Most human trials investigate spirulina or chlorella separately, and rigorous head-to-head comparisons or factorial designs testing both in combination are scarce. Consequently, it is not currently possible to conclude whether combined use produces additive or synergistic benefits beyond those achievable with either organism alone. Another practical limitation is product heterogeneity: combined formulations vary markedly in species/strain, processing (cell-wall disruption), extract standardization (e.g., phycocyanin content), and contaminant control, which can confound outcomes and safety interpretation.

From a translational perspective, we propose that combined supplementation be framed as a hypothesis-driven strategy warranting targeted clinical testing. Future randomized controlled trials should compare (i) spirulina alone, (ii) chlorella alone, and (iii) combined supplementation, ideally with standardized products and multi-omic biomarker panels (metabolomics, inflammatory proteomics, gut microbiome profiling) to identify mechanistic signatures and responder phenotypes. Such designs would clarify whether co-supplementation improves integrated endpoints (lipid profile, insulin sensitivity, blood pressure, inflammatory markers, oxidative stress biomarkers) more effectively than monotherapy.

In summary, combining chlorella and spirulina is mechanistically plausible due to complementary bioactive profiles and partially distinct dominant pathways (intracellular metabolic/redox signaling vs. gut–immune modulation and detox-associated effects). Nevertheless, the current lack of robust clinical trials directly testing combined regimens prevents definitive conclusions regarding superiority over single-alga supplementation. Well-controlled comparative and combination studies remain a priority to establish evidence-based recommendations. At present, this concept should be considered exploratory, as robust clinical evidence directly evaluating combined supplementation strategies remains lacking.

## 8. Healthy Aging Perspective

Aging is characterized by the progressive accumulation of molecular and cellular damage, mitochondrial dysfunction, altered nutrient sensing, impaired proteostasis, stem cell exhaustion, and chronic low-grade inflammation, collectively described as the “hallmarks of aging”. Among these, mitochondrial dysfunction and inflammaging occupy central positions linking metabolic disease, cardiovascular pathology, neurodegeneration, and immune dysregulation. The broad biological activity of *Chlorella* and *Arthrospira* (spirulina) suggests potential relevance in modulating several of these interconnected processes [4,5,75].

Mitochondrial dysfunction during aging is associated with increased reactive oxygen species production, reduced oxidative phosphorylation efficiency, impaired mitophagy, and altered AMPK–mTOR signaling. Preclinical evidence indicates that spirulina-derived phycocyanin can reduce mitochondrial oxidative stress, enhance antioxidant enzyme expression via Nrf2/HO-1 activation, and improve metabolic signaling through AMPK-dependent mechanisms. Given that AMPK is a core energy sensor influencing mitochondrial biogenesis and autophagy, spirulina’s activation of this pathway may contribute to improved mitochondrial turnover and cellular stress adaptation [17,27,37,38,76].

*Chlorella*’s contribution to aging-related pathways appears to be mediated predominantly through redox stabilization, immune modulation, and gut–systemic interactions. By reducing NF-κB-driven inflammatory signaling and enhancing antioxidant enzyme activity, chlorella may attenuate the pro-inflammatory milieu characteristic of aging tissues. Furthermore, its influence on gut microbiota composition and short-chain fatty acid production suggests a hypothetical role in modulating the gut–immune–brain axis—a pathway increasingly recognized in healthy aging research, though direct human evidence linking chlorella supplementation to brain health outcomes is currently lacking [21,26,77].

The concept of “inflammaging”—persistent, low-grade systemic inflammation driven by metabolic and immune dysregulation—is particularly relevant. Both microalgae have demonstrated capacity to reduce circulating CRP and pro-inflammatory cytokines in clinical populations with metabolic syndrome and type 2 diabetes. Although aging-specific trials are lacking, the combined antioxidant, anti-inflammatory, and metabolic benefits support a theoretical role in promoting metabolic resilience and reducing biological age-associated stress burden [23,33,34,57].

Neuroprotective implications are also plausible. Oxidative stress and microglial activation are central drivers of cognitive decline and neurodegenerative disorders. Spirulina-derived phycocyanin exhibits anti-inflammatory and anti-apoptotic activity in experimental neural models, while chlorella polysaccharides have shown neuroprotective potential in preclinical settings. Whether these effects translate into measurable cognitive benefits in humans remains an open question requiring well-designed longitudinal studies [27,38,63,78].

Aging interventions must, however, be evaluated not solely by their capacity to reduce isolated biomarkers, but by their impact on systemic resilience—the ability to respond to metabolic, oxidative, and inflammatory stressors. From this perspective, both chlorella and spirulina function as complex biological agents rather than simple antioxidant supplements. Their multi-target activity aligns conceptually with systems-based approaches to healthy longevity [5,79].

However, the absence of long-term human studies assessing frailty, functional decline, mitochondrial biomarkers, or epigenetic aging clocks represents a major research gap. Until such data are available, claims regarding direct longevity extension remain speculative [5,80]. Therefore, while mechanistic plausibility is strong, conclusions regarding direct effects on human aging trajectories remain speculative in the absence of long-term clinical studies incorporating validated aging biomarkers.

## 9. Clinical Relevance and Positioning Among Nutraceutical Strategies

Nutraceutical interventions have gained increasing attention as complementary strategies for the prevention and management of cardiometabolic and inflammatory disorders [5,7,57]. The clinical value of *Chlorella* and *Arthrospira* (spirulina) should therefore be interpreted within the broader landscape of nutraceutical approaches targeting these conditions.

Unlike single-molecule supplements, such as isolated antioxidants or purified polyphenols, microalgae represent complex biological matrices containing proteins, pigments, lipids, polysaccharides, and micronutrients that act in concert. This compositional complexity likely contributes to their multi-target physiological effects, but it also complicates standardization and direct comparison with pharmacological agents [4,5,57,81,82].

### 9.1. Positioning in Dyslipidemia and Metabolic Syndrome

Among the nutraceuticals used in dyslipidemia, agents such as red yeast rice (monacolin K), plant sterols, berberine, and omega-3 fatty acids have demonstrated measurable lipid-lowering effects. Spirulina consistently shows modest but statistically significant reductions in total cholesterol (approximately 10–20 mg/dL), LDL-C (approximately 8–15 mg/dL), and triglycerides (approximately 15–30 mg/dL) across meta-analyses of randomized controlled trials. The magnitude of lipid reduction is generally smaller than that observed with statins or red yeast rice but comparable to that of some plant-derived supplements. Importantly, spirulina’s lipid effects are accompanied by reductions in oxidative stress and inflammatory markers, suggesting a broader cardiometabolic impact beyond simple LDL lowering [7,9,57,83].

*Chlorella* demonstrates lipid improvements in some studies, but its signal appears less consistent than that of spirulina. However, chlorella’s blood pressure lowering effects and enhancement of antioxidant capacity may provide additional cardiovascular benefits not fully captured by lipid endpoints alone. Thus, spirulina may be positioned as the stronger candidate for dyslipidemia-focused interventions, whereas chlorella may have a more balanced profile in individuals with combined prehypertension and oxidative stress burden [8,9,81,84].

### 9.2. Glycemic Control and Insulin Resistance

Compared with established nutraceuticals such as berberine—known for relatively robust glucose-lowering effects—spirulina’s impact on fasting glucose and HOMA-IR appears moderate. Nevertheless, improvements are reproducible across several small-to-medium-sized trials, particularly in patients with type 2 diabetes or metabolic syndrome. These effects may reflect AMPK activation and reduced inflammatory interference with insulin receptor signaling [9,18,57,85].

*Chlorella* has demonstrated improvements in glycemic markers in some cohorts, though the evidence base is smaller. It may be particularly relevant in patients with metabolic dysfunction accompanied by increased oxidative stress or hepatic enzyme abnormalities [15,41,86].

Neither alga currently rivals pharmacological glucose-lowering agents; however, their safety profile and systemic anti-inflammatory activity may support their role as adjunctive interventions [5,87].

### 9.3. Blood Pressure and Endothelial Function

In the domain of blood pressure regulation, chlorella demonstrates relatively consistent reductions in systolic blood pressure in prehypertensive individuals. The mechanism likely involves improved endothelial redox balance and reduced inflammatory signaling rather than direct vasodilatory action. Spirulina has also shown modest antihypertensive effects in some trials, but the evidence appears slightly stronger for chlorella in this specific endpoint [8,9,41,57,88].

When compared with established interventions such as omega-3 fatty acids or dietary nitrate sources, the antihypertensive magnitude remains moderate. However, combined lipid, inflammatory, and blood pressure modulation may provide cumulative vascular benefit.

### 9.4. Anti-Inflammatory and Redox Positioning

Chronic low-grade inflammation is a common denominator across cardiometabolic and aging-related disorders. Curcumin, omega-3 fatty acids, and certain polyphenols are frequently discussed in this context. Spirulina’s reduction of CRP and modulation of NF-κB–linked pathways place it among nutraceuticals with documented systemic anti-inflammatory activity. *Chlorella*, while less studied in large meta-analyses, consistently enhances antioxidant enzyme capacity and reduces oxidative markers in clinical contexts [17,33,34,57,89].

Unlike high-dose isolated antioxidants, which may blunt physiological redox signaling, microalgae appear to act by enhancing endogenous antioxidant systems (e.g., Nrf2 activation), aligning more closely with adaptive stress–response modulation rather than simple radical scavenging.

### 9.5. Comparative Clinical Strength, Safety and Special Populations

#### 9.5.1. Detoxification and Environmental Stress Contexts

One distinctive niche where chlorella may offer added value is environmental stress exposure. Preclinical models demonstrate heavy metal binding and attenuation of toxicant-induced oxidative damage. While large-scale human detoxification trials are lacking, this feature differentiates chlorella from many other nutraceuticals and may justify its use in specific exposure contexts [11,12,26,81,90].

Spirulina has less documented heavy metal binding activity but may mitigate oxidative consequences of environmental stress through intracellular redox modulation.

#### 9.5.2. Comparative Clinical Strength

From an evidence hierarchy perspective:

Spirulina: stronger volume of RCTs and meta-analyses; clearer lipid and CRP signal; stronger mechanistic mapping to AMPK/NF-κB [7,8,9,23,34,48,57,81].

*Chlorella*: fewer high-powered RCTs; stronger signal in blood pressure modulation and detox-related contexts; notable gut–immune interactions in preclinical data [91].

Neither alga should be considered a replacement for pharmacotherapy in high-risk patients. Rather, they may serve as adjunctive strategies within comprehensive lifestyle interventions that include dietary optimization, physical activity, and weight management.

#### 9.5.3. Safety, Drug Interactions and Practical Use

Safety data are reassuring: both organisms are well-tolerated at the doses studied, with mild gastrointestinal symptoms accounting for most reported adverse effects. The primary safety concern lies not in intrinsic toxicity but in product contamination—specifically heavy metal accumulation and cyanotoxin co-contamination—and the absence of standardized quality benchmarks. Clinical recommendations must therefore emphasize verified, tested preparations from controlled production systems. Several additional safety considerations warrant attention in clinical contexts. Spirulina has a high vitamin K content that may interact with anticoagulant therapy (e.g., warfarin), requiring monitoring in patients on anticoagulants. Its high protein load may be relevant in patients with phenylketonuria (PKU) or advanced renal insufficiency, where high-protein supplementation is contraindicated. Both microalgae may exert immunostimulatory effects through polysaccharide fractions, which could theoretically be problematic in immunosuppressed individuals or those with autoimmune conditions; however, clinical evidence on this interaction remains limited. Pregnant and breastfeeding women should use these supplements only under medical supervision due to insufficient safety data in these populations. Consequently, these microalgae should be viewed as adjunctive interventions rather than primary therapeutic strategies, particularly in high-risk or clinically advanced patient populations.

## 10. Knowledge Gaps and Future Directions

Despite growing mechanistic and clinical evidence, several fundamental limitations currently constrain the translational interpretation and clinical applicability of findings related to chlorella and spirulina.

A key limitation across the reviewed literature concerns variability in supplement composition, cultivation conditions, processing methods, and formulation (whole biomass vs. extract vs. isolated fraction), all of which substantially affect bioavailability and biological potency. Differences in cell wall integrity, drying methods (spray-drying vs. freeze-drying), and particle size further contribute to inter-study heterogeneity. Future research should prioritize chemically characterized and standardized preparations to enable meaningful cross-study comparisons and dose–response analyses.

First, product heterogeneity remains a fundamental issue. Strain differences, cultivation conditions, harvesting methods, cell-wall disruption techniques, and extraction protocols significantly influence bioactive composition. In many clinical trials, detailed compositional characterization is lacking, limiting reproducibility and mechanistic interpretation. Future studies should incorporate standardized phytochemical profiling and batch-level quality control [1,2,11,12,92].

Second, dose–response relationships remain insufficiently defined. Most clinical trials use doses between 1–8 g/day, yet optimal dosing for distinct outcomes (lipid lowering, glycemic control, immune modulation) has not been systematically established. Controlled dose-escalation studies are needed [4,9,18,48,57].

Third, direct head-to-head comparisons between chlorella and spirulina are virtually absent. Most trials evaluate each alga independently, preventing robust conclusions regarding relative efficacy. Given their partially distinct biological profiles—spirulina with stronger evidence for lipid modulation and intracellular signaling activation, and chlorella with prominent antioxidant, blood pressure, and gut–immune effects—comparative trials are warranted [5,7,8,9,48,57,81,93].

Fourth, combination strategies remain underexplored. Although theoretical synergy is biologically plausible, with protein-rich spirulina providing signaling modulation and AMPK activation while chlorella contributes polysaccharide-mediated immune regulation and chlorophyll-associated antioxidant support, controlled clinical trials evaluating co-supplementation are lacking. Future randomized studies should examine additive or synergistic effects on integrated cardiometabolic endpoints [5,48,81,94].

Fifth, aging-specific endpoints remain entirely absent from clinical trial designs. Meaningful assessment of microalgae supplementation in the context of aging would require the incorporation of mitochondrial respiration assays, epigenetic clock analysis (e.g., Horvath or GrimAge clocks), frailty indices, and longitudinal tracking of inflammatory proteomics. Until such endpoints are incorporated into clinical trial designs, extrapolation to aging-related outcomes will remain speculative [3,9,45].

In addition, safety and contamination monitoring require continued vigilance. Heavy metal accumulation and potential cyanotoxin contamination remain concerns in poorly controlled production systems, underscoring the importance of rigorous quality assurance [11,12].

An emerging and underexplored avenue concerns the chemopreventive potential of both microalgae. Converging in vivo evidence from independent animal models indicates antineoplastic activity for both organisms. Dietary administration of *Chlorella pyrenoidosa* at 3% suppressed tumor frequency by 61% and extended tumor latency by 12.5 days in NMU-induced mammary carcinogenesis in rats, with immunohistochemical evidence of increased caspase-7 expression and reduced VEGFR-2 [95]. In a parallel model, Spirulina (*Arthrospira platensis*) supplementation reduced the incidence of DMBA-induced rat mammary tumors from 87% to 13%, accompanied by reduced Ki-67 and estrogen receptor-α expression in tumor tissue, and in vitro induction of p53–p21-mediated cell cycle arrest and Bax/Bcl-2-regulated apoptosis in MCF-7 cells [96]. These parallel in vivo findings for both organisms constitute a qualitatively stronger level of evidence than in vitro data alone, and establish a shared chemopreventive trajectory warranting further mechanistic characterization. Complementing this, preliminary in vitro evidence indicates that a water extract of commercial Spirulina (*Arthrospira platensis*) exerts antiproliferative and pro-apoptotic effects in human non-small-cell lung carcinoma A549 cells, involving G1 phase arrest, reduced Akt/Rb phosphorylation, decreased cyclin D1 and CDK4 expression, and an increased Bax/Bcl-2 ratio, without cytotoxic effects on normal fibroblasts [97]. Similarly, a water extract of *Chlorella pyrenoidosa* has been shown to inhibit metabolic activity, DNA synthesis, and the migratory capacity of human endometrial adenocarcinoma cell lines (HEC-1-B, KLE, EDC), with concurrent induction of apoptosis and membrane damage [98]. Extending the chemopreventive evidence to additional cancer types and treatment strategies, water extracts of chlorella combined with young green barley showed synergistic antiproliferative activity against human breast adenocarcinoma T47D cells [99] and enhanced chemopreventive effects against colon cancer cell lines HT-29 in vitro [100], with no toxicity toward normal epithelial cells in either model. Taken together, these findings indicate that both organisms harbor bioactives with oncological relevance beyond their established cardiometabolic properties. Translation to further in vivo models and ultimately clinical contexts will require systematic mechanistic characterization and dose–response optimization. It should be emphasized that current evidence for anticancer effects is largely limited to preclinical models, and no robust clinical data currently support therapeutic efficacy in humans.

Finally, potential publication bias and small-study effects should be considered, as positive findings are more likely to be reported in nutraceutical research, potentially leading to the overestimation of effect sizes. Addressing these limitations will be essential for advancing microalgae research from exploratory and adjunctive use toward evidence-based clinical application.

## 11. Conclusions

The available evidence suggests that both *Chlorella* and *Arthrospira* (spirulina) exert broad biological activities including antioxidant, anti-inflammatory, metabolic, and immunomodulatory effects. Spirulina currently demonstrates stronger clinical evidence for lipid profile improvement and systemic inflammation reduction, whereas chlorella appears particularly promising for blood pressure regulation, enhancement of antioxidant defenses, and gut-microbiota-related effects. Rather than representing competing interventions, these microalgae appear to provide complementary biological activities that may support integrated strategies for cardiometabolic health and healthy aging. We propose that future research prioritize standardized preparations, mechanistically informed clinical trials, and multi-omics approaches to clarify dose–response relationships and identify responder phenotypes.

Taken together, the available evidence supports a model in which chlorella and spirulina function as multi-axis regulators of interconnected redox, inflammatory, and metabolic pathways. Rather than acting as single-compound nutraceuticals, their biological effects emerge from the coordinated modulation of complex signaling networks. This systems-level perspective provides a conceptual framework for future research aimed at defining their clinical utility, optimizing intervention strategies, and identifying responder phenotypes through multi-omics approaches.

## Figures and Tables

**Table 1:** Major bioactive constituents of *Chlorella* and *Arthrospira* (spirulina): composition, comparative profile, and selected references [1,2,3,10,11].

Bioactive Class	Spirulina (*Arthrospira*)	*Chlorella*	Comparative Notes
Protein content (% dry weight)	~60–70%	~50–60%	Spirulina generally shows higher protein density
Phycobiliproteins (C-phycocyanin)	High (major characteristic pigment)	Absent	Distinct advantage of spirulina
Chlorophyll content	Moderate	High (chlorophyll a and b)	*Chlorella* richer source
Carotenoids (β-carotene, lutein, zeaxanthin)	Present	Present (often higher lutein)	Both significant but with different profiles
Polysaccharides	Sulfated polysaccharides; immunomodulatory fractions	Cell-wall polysaccharides and bioactive fractions	Structural differences; gut-related effects reported for *Chlorella*
Lipids (% dry weight)	~6–8%	~5–15%	*Chlorella* may contain higher total lipid fraction; values vary considerably by strain and cultivation conditions
γ-Linolenic acid (GLA)	Present	Minimal or absent	Advantage of spirulina
Nucleotides/CGF fraction	Not characteristic	Present (*Chlorella* Growth Factor concept)	Characteristic feature of *Chlorella*
Cell wall complexity	Lower cellulose content	Rigid cell wall	Processing important for bioavailability
Minerals (Fe, Mg, K)	High iron bioavailability	High mineral content	Both nutritionally dense

**Table 2:** Representative clinical studies evaluating the effects of *Chlorella* and spirulina supplementation on cardiometabolic and oxidative stress parameters.

Study (Author, Year)	Population	Intervention	Study Design	Outcomes	Key Findings	Effect Direction
Mazokopakis et al., 2014 [44]	NAFLD	Spirulina 6 g/day, 6 months	Open-label	Lipids, liver enzymes	↓ alanine aminotransferase (ALT), ↓ triglycerides (TG), improved steatosis	↓ lipids; ↓ liver enzymes
Serban et al., 2016 [46]	Dyslipidemia	Spirulina 1–10 g/day	Meta-analysis	Lipid profile	↓ TC, ↓ LDL-C, ↓ TG	↓ lipids
Lee et al., 2008 [35]	Type 2 diabetes	Spirulina 8 g/day, 12 weeks	RCT	Glycemia, lipids	Improved lipid profile and glycemia	↓ lipids; ↓ glycemia
Hernández-Lepe et al., 2019 [25]	Metabolic syndrome	Spirulina 4.5 g/day, 6 weeks	RCT	Oxidative stress	↓ MDA, ↑ antioxidant enzymes	↓ oxidative stress; ↑ antioxidants
Rezaiyan et al., 2023 [19]	Type 2 diabetes	Spirulina 2 g/day, 8 weeks	DB-RCT	Weight, CRP	↓ CRP, ↓ body weight	↓ inflammation; ↓ weight
Hamedifard et al., 2019 [47]	Dyslipidemia	Spirulina	Meta-analysis	Lipid profile	↓ LDL-C, ↓ TG	↓ lipids
Fallah et al., 2018 [8]	Hypertension	*Chlorella* 2 g/day, 12 weeks	RCT	Blood pressure	↓ SBP, ↓ DBP	↓ blood pressure
Rajabzadeh Karizi et al., 2023 [18]	Type 2 diabetes	Spirulina 2 g/day, 8 weeks	RCT	Glycemia, oxidative stress	↓ glucose, ↓ lipid peroxidation	↓ glycemia; ↓ oxidative stress
Fu et al., 2025 [48]	Overweight	Spirulina 2 g/day, 12 weeks	RCT	Lipids, insulin resistance	↓ TG, improved insulin sensitivity	↓ lipids; ↑ insulin sensitivity
Ebrahimi-Mameghani et al., 2017 [41]	Prehypertension	*Chlorella* 3 g/day, 12 weeks	RCT	BP, antioxidant status	↓ SBP, ↑ antioxidant capacity	↓ blood pressure; ↑ antioxidants
Rajabzadeh Karizi et al., 2023 [18]	Type 2 diabetes	Spirulina 2 g/day, 12 weeks	DB-RCT	Lipids, glycemia	Improved lipid profile and glycemia	↓ lipids; ↓ glycemia
Moradi et al., 2019 [49]	Obesity	Spirulina	Meta-analysis	Weight, body mass index (BMI)	Modest ↓ body weight and BMI	↓ weight; ↓ BMI

**Table 3:** Mechanistic studies supporting the biological activity of *Chlorella* and *Arthrospira* (spirulina) in experimental models.

Study (Author, Year)	Species/Bioactive Fraction	Model	Key Pathways/Targets	Main Readouts	Core Mechanistic Conclusion
Byun et al., 2015 [24]	*Chlorella vulgaris* extract	Hepa1c1c7 cells (in vitro)	Nrf2-associated Phase II response	Quinone reductase activity, cytoprotection	*Chlorella* extract induced antioxidant Phase II defenses via Nrf2-related signaling.
Sibi et al., 2016 [15]	*Chlorella vulgaris* fractions	Inflammatory cell model (in vitro)	NF-κB, iNOS, COX-2	NO production, inflammatory mediators	*Chlorella* fractions suppressed pro-inflammatory mediators linked to NF-κB signaling.
Farag et al., 2023 [26]	*Chlorella vulgaris*	CdCl_2_ toxicity in rats (in vivo)	Nrf2/NF-κB axis	Oxidative stress markers, tissue injury	*Chlorella* reduced oxidative damage through modulation of Nrf2 and NF-κB pathways.
Velankanni et al., 2023 [21]	*Chlorella vulgaris* biomass	DSS colitis mouse model	Gut–immune axis	Microbiota composition, SCFA, Treg cells	*Chlorella* altered microbiota and increased SCFA production, attenuating intestinal inflammation.
Wang et al., 2025 [61]	*Chlorella pyrenoidosa* polysaccharides	Alzheimer’s disease model	Neuroinflammation pathways	APP/tau markers, inflammatory mediators	Peptides showed neuroprotective effects through reduction in neuroinflammation.
Pi et al., 2025 [52]	CPP-3a polysaccharide (*C. pyrenoidosa*)	Macrophage model (in vitro)	TLR4/2–MyD88–NF-κB, p38 MAPK	p65 nuclear translocation, M1 markers	Polysaccharide acted as an immunomodulator via TLR4-dependent signaling pathways.
Cherng et al., 2007 [62]	C-phycocyanin	RAW264.7 macrophages (in vitro)	NF-κB, iNOS	NO production, TNF-α	Phycocyanin suppressed inflammatory mediators in activated macrophages.
Liu et al., 2020 [17]	Phycocyanin	Oxidative injury model (in vivo)	Nrf2/HO-1 pathway	Oxidative stress markers	Phycocyanin alleviated oxidative damage via activation of Nrf2-dependent antioxidant responses.
Liu et al., 2022 [27]	Phycocyanin peptide	Pulmonary fibrosis model	Keap1–Nrf2–HO-1	Oxidative and inflammatory injury markers	Peptide reduced fibrosis-related oxidative damage through Nrf2 pathway activation.
Hao et al., 2022 [37]	Phycocyanin	Diet-induced diabetes model	AMPK, AKT signaling	Insulin sensitivity, metabolic markers	Spirulina pigment improved metabolic signaling and reduced insulin resistance.
Althobaiti et al., 2024 [39]	*Arthrospira platensis* nanoparticles	Diabetic nephropathy model	NRF2/HO-1, NF-κB	Renal oxidative stress and inflammation	Spirulina nanoparticles protected kidney tissue via NRF2 activation and NF-κB inhibition.
Li et al., 2023 [63]	C-phycocyanin	Mouse liver injury model	Gut–liver axis	Microbiota composition, liver injury markers	Phycocyanin improved liver injury partly through microbiota-mediated mechanisms.

**Table 4:** Comparative biological profile of *Chlorella* and *Arthrospira* (spirulina): mechanisms and clinical evidence.

Biological Domain	Spirulina (*Arthrospira*)	*Chlorella*	Comparative Interpretation
Major bioactive components	Phycocyanin, phycocyanobilin, peptides, γ-linolenic acid (GLA), high protein content	Chlorophylls, carotenoids, polysaccharides, CGF (nucleotide/peptide-rich fraction)	Spirulina characterized by pigment-driven signaling molecules; chlorella richer in chlorophyll and structural polysaccharides
Redox modulation	Strong evidence for Nrf2 activation and increased antioxidant enzyme activity	Consistent enhancement of antioxidant capacity and detox-associated redox stabilization	Both algae demonstrate antioxidant activity; spirulina is more mechanistically linked to intracellular signaling
Inflammatory signaling	Inhibition of NF-κB and reduced pro-inflammatory mediators in multiple models; CRP reduction in RCTs	Modulation of inflammatory mediators and immune signaling; gut-mediated immune effects	Spirulina has stronger clinical inflammation data; chlorella contributes through the immune and gut pathways
Metabolic signaling	Activation of AMPK and AKT pathways in metabolic disease models	Limited direct evidence for AMPK activation	Spirulina shows clearer mechanistic links to intracellular metabolic regulation
Lipid metabolism	Consistent reductions in LDL-C and triglycerides across meta-analyses	Modest lipid improvements in smaller trials	Spirulina demonstrates stronger clinical evidence for dyslipidemia
Glycemic control	Improvements in fasting glucose and HOMA-IR in T2DM and metabolic syndrome	Moderate improvements reported in metabolic dysfunction contexts	Slight advantage for spirulina
Blood pressure regulation	Mild antihypertensive effects in some studies	Consistent reductions in systolic blood pressure in prehypertensive subjects	*Chlorella* may have stronger vascular pressure effects
Gut microbiota modulation	Emerging evidence for microbiota interaction	Demonstrated microbiota reshaping and increased SCFA production in experimental models	*Chlorella* shows stronger gut-mediated activity
Detoxification potential	Limited direct evidence	Demonstrated heavy metal binding and reduction in toxicant-induced oxidative stress	*Chlorella* advantage in detox-related contexts
Neuroprotective potential	Phycocyanin shows anti-inflammatory and anti-apoptotic effects in neural models	Polysaccharide fractions demonstrate neuroprotective activity in experimental models	Both promising but limited human evidence
Mitochondrial support	Evidence for reduced mitochondrial ROS and AMPK-linked metabolic effects	Indirect mitochondrial support via redox stabilization	Spirulina mechanistically clearer
Clinical evidence volume	Larger number of RCTs and meta-analyses	Smaller number of high-quality clinical trials	Spirulina currently has stronger clinical evidence base
Potential complementarity	Intracellular signaling and metabolic modulation	Gut–immune and detox-associated effects	Biological complementarity suggests potential benefit of combined supplementation

## Data Availability

No new data were created or analyzed in this study. Data sharing is not applicable to this article.

## Abbreviations

AKT	Protein kinase B
ALT	Alanine aminotransferase
AMPK	AMP-activated protein kinase
BMI	Body mass index
CGF	*Chlorella* Growth Factor
CRP	C-reactive protein
DBP	Diastolic blood pressure (DBP)
GLA	Gamma-linolenic acid
GPx	Glutathione peroxidase
HO-1	Heme oxygenase-1
HOMA-IR	Homeostatic model assessment of insulin resistance
IL	Interleukin
LDL-C	Low-density lipoprotein cholesterol
MAPK	Mitogen-activated protein kinase
MDA	Malondialdehyde
mTOR	Mechanistic target of rapamycin
NAFLD	Non-alcoholic fatty liver disease
NF-κB	Nuclear factor kappa B
NK	Natural killer
NO	Nitric oxide
RCT	Randomized controlled trial
ROS	Reactive oxygen species
SBP	Systolic blood pressure
SCFA	Short-chain fatty acids
SOD	Superoxide dismutase
TAC	Total antioxidant capacity
TG	Triglycerides
TLR	Toll-like receptor
TNF-α	Tumor necrosis factor-alpha
